# Sphingolipid metabolic subtyping defines SPHK1 as an oncogene and characterizes immunosuppressive TME features in gastric cancer

**DOI:** 10.1186/s12935-026-04401-4

**Published:** 2026-07-27

**Authors:** Zeyu Wang, Zhuying Yue, Chengkai Jiang

**Affiliations:** 1 Joint Laboratory of Biomedical Artificial Intelligence, Shanghai East Hospital, Tongji University School of Medicine, 200120 Shanghai, China; 2 State Key Laboratory of Systems Medicine for Cancer, Shanghai Cancer Institute, Renji Hospital Affiliated to Shanghai Jiao Tong University school of Medicine, Shanghai, 200127 China; 3 Shanghai Key Laboratory of Cancer Systems Regulation and Clinical Translation (CSRCT-SHANGHAI), Jiading District Central Hospital, Shanghai, 201800 China; 4 Shanghai Key Laboratory of Cancer Systems Regulation and Clinical Translation, Jiading District Central Hospital Affiliated Shanghai University of Medicine & Health Sciences, Shanghai, 201800 China

**Keywords:** Sphingolipid metabolism-related genes (SMGs), SPHK1, Gastric cancer (GC), Metabolic-immune crosstalk

## Abstract

Gastric cancer (GC) exhibits profound heterogeneity and poor clinical outcomes, underscoring the need for improved prognostic stratification and deeper insights into metabolism-immune interactions within the tumor microenvironment (TME). Although sphingolipid metabolic reprogramming is known to influence TME remodeling, systematic GC subtyping based on sphingolipid metabolism-related genes (SMGs) and the spatial regulatory roles of key metabolic enzymes remain insufficiently characterized. Using TCGA RNA sequencing data, we performed unsupervised clustering of SMGs and identified two metabolic subtypes. Cluster A was marked by upregulated sphingolipid metabolism, extensive extracellular matrix remodeling, and enrichment of immunosuppressive pathways, corresponding to significantly worse survival. Cluster B showed enrichment of energy metabolic programs and increased inflammatory immune infiltration, aligning with a more favorable prognosis. Spatial transcriptomic analysis revealed SPHK1 enrichment at the tumor invasive front, where high SPHK1 expression correlated with increased dendritic cell and neutrophil infiltration but not CD8^+^ T cell accumulation, suggesting a myeloid-dominant immunosuppressive niche. Functionally, SPHK1 knockdown suppressed GC cell proliferation and migration, increased E-cadherin, decreased mesenchymal markers, and inhibited TGF-β1 signaling, confirming its role in EMT and tumor progression. Collectively, these findings identify sphingolipid metabolic remodeling and aberrant SPHK1 activation as key drivers of GC immune evasion and potential therapeutic vulnerabilities.

## Introduction

Gastric cancer (GC) is a major global health problem and remains one of the most prevalent and lethal malignancies worldwide [1, 2]. It ranks fifth in incidence and third in cancer-related mortality, with the greatest disease burden observed in East Asia [3–5]. Most patients are diagnosed at an advanced stage, and five-year survival rates remain poor, typically below 25–30% [6, 7]. Despite advances in surgery, chemotherapy, and targeted therapy, recurrence and metastasis remain frequent, and the prognosis of advanced GC has not improved substantially [8, 9]. This persistent clinical challenge underscores an urgent need to identify novel biomarkers and therapeutic targets that could improve early diagnosis and treatment outcomes [10].

Metabolic reprogramming has emerged as a hallmark of cancer and plays a crucial role in supporting uncontrolled cell proliferation and survival [11–13]. In gastric cancer, dysregulated lipid metabolism has gained increasing attention as a driver of malignant progression [14, 15]. Metabolomic and transcriptomic analyses have revealed that alterations in lipid metabolic pathways, particularly sphingolipid metabolism, are associated with tumor aggressiveness and patient prognosis [16, 17]. Sphingolipids are a family of bioactive lipids that play central roles in regulating cell fate, and the genes involved in their metabolic regulation are collectively referred to as sphingolipid metabolism-related genes (SMGs) [18–20]. The balance between ceramide and sphingosine, which promote apoptosis, and sphingosine-1-phosphate (S1P), which promotes proliferation, survival, and angiogenesis, is referred to as the sphingolipid rheostat [21, 22]. In many cancers, this balance shifts toward S1P, thereby facilitating tumor growth, invasion, and resistance to therapy [23–26]. Enzymes that regulate sphingolipid metabolism are therefore central regulators of cancer cell behavior, affecting proliferation, metastasis, and immune escape [27]. However, the specific contribution of sphingolipid metabolic enzymes to gastric carcinogenesis remains incompletely understood [28].

Among these enzymes, sphingosine kinase 1 (SPHK1) is of particular interest because of its central role in converting sphingosine to S1P and thereby promoting a pro-survival signaling state [29, 30]. SPHK1 is frequently overexpressed in various human malignancies and is associated with tumor aggressiveness and poor clinical outcomes [31–33]. In gastric cancer, elevated SPHK1 expression has been detected in both tumor tissues and established cell lines, and patients with higher SPHK1 expression exhibit shorter overall survival and more advanced disease stages [34–36]. Mechanistic studies indicate that SPHK1 promotes oncogenic progression through several downstream pathways, including activation of PI3K/AKT and NF-κB signaling, which enhance proliferation, inhibit apoptosis, and facilitate invasion [37–40]. Moreover, SPHK1 overexpression contributes to epigenetic remodeling and has been associated with hypermethylation and silencing of tumor suppressor genes such as STAT1 [36]. This mechanism further supports the role of SPHK1 as a critical oncogenic driver and a potential therapeutic target in gastric cancer [41, 42].

In this study, we performed a comprehensive investigation of sphingolipid metabolism-related genes in gastric cancer by integrating bioinformatic analyses of patient datasets with in vitro functional validation. Transcriptomic profiling identified SPHK1 as one of the most markedly upregulated sphingolipid metabolism-related genes in gastric cancer. We further examined the biological consequences of SPHK1 knockdown in two independent gastric cancer cell lines, in which SPHK1 silencing significantly suppressed cell proliferation, migration, and survival, indicating its essential role in maintaining the malignant phenotype of gastric cancer cells. Subsequent analyses explored signaling pathways associated with SPHK1 activity. Although this study was limited to in vitro experiments without in vivo validation, these findings provide strong mechanistic evidence that SPHK1 contributes to gastric cancer progression and highlight sphingolipid metabolism as a promising target for future therapeutic development.

## Materials and methods

### Computational analysis of sphingolipid metabolism genes using R-based bioinformatics pipelines

Publicly available gastric cancer gene expression datasets (GSE54129 and GSE79973) were obtained from the NCBI GEO database. Raw CEL files were background-corrected, normalized using the robust multi-array average (RMA) algorithm, and summarized at the gene level in R using Bioconductor packages. A curated list of sphingolipid metabolism-related genes (SMGs) was compiled from MSigDB and covered genes involved in biosynthesis, degradation, and signaling. Differential expression analysis between tumor and normal samples was performed using the limma package with empirical Bayes moderation and Benjamini-Hochberg correction; genes meeting FDR < 0.05 and the prespecified |log_2_FC| thresholds were considered significant. SMG-based unsupervised clustering using Ward’s method with Euclidean distances calculated from z-scored expression profiles was performed to classify tumors into two molecular subtypes, which were visualized using heatmaps and principal component analysis (PCA). Gene set variation analysis (GSVA) was used to infer pathway activity across subtypes, whereas immune infiltration was assessed using ssGSEA and ESTIMATE. Mutation and copy-number data, when available, were visualized using oncoplots. Kaplan-Meier survival curves, log-rank tests, and Cox regression models were applied to evaluate the prognostic relevance of SPHK1 and SMG-defined clusters. Functional enrichment analysis of subtype-specific or SPHK1-associated genes was performed using clusterProfiler for Gene Ontology (GO) terms. All statistical analyses were conducted in R, and statistical significance was defined as FDR < 0.05 unless otherwise specified.

### Validation and functional characterization of SPHK1 through online bioinformatics platforms

SPHK1 expression and associated functional features in GC were analyzed using multiple online bioinformatics platforms. mRNA expression levels of SPHK1 in gastric tumor and adjacent normal tissues were analyzed using GEPIA (http://gepia.cancer-pku.cn), UALCAN (http://ualcan.path.uab.edu), and TNMplot (http://tnmplot.com), and boxplots were generated to visualize differential expression. Protein-level distribution of SPHK1 was evaluated using immunohistochemistry data from the Human Protein Atlas (https://www.proteinatlas.org). The GSCALite database (http://bioinfo.life.hust.edu.cn/web/GSCALite) was used to assess SPHK1-related pathway activity, copy-number variation, promoter methylation, and drug sensitivity, and the results were visualized as pathway activity scores, CNV frequency plots, methylation correlation plots, and drug response heatmaps. A protein-protein interaction network centered on SPHK1 was constructed using STRING (https://string-db.org) to identify predicted interacting partners. Potential regulatory interactions involving SPHK1, including candidate microRNAs, were predicted using muTarget database (https://mubiogate.com/mutarget). Immune infiltration and immune checkpoint associations were examined using TIMER (https://cistrome.shinyapps.io/timer) and TISIDB (http://cis.hku.hk/TISIDB), whereas TIDE (http://tide.dfci.harvard.edu) was applied to evaluate SPHK1-related T-cell dysfunction and exclusion within the gastric tumor immune microenvironment. Single-cell localization of SPHK1 within the gastric tumor microenvironment was visualized using TISCH2 (http://tisch.comp-genomics.org) with cell type-specific UMAP plots, and spatial transcriptomic patterns were characterized using SpatialDB (https://www.spatialomics.org/SpatialD). All analyses were performed to generate publication-quality visualizations.

### Cell culture and SPHK1 knockdown

Human gastric cancer cell lines AGS and HGC27 were maintained in Dulbecco’s Modified Eagle Medium (DMEM; Invitrogen) supplemented with 10% fetal bovine serum (FBS; Gibco) at 37 °C in a humidified incubator containing 5% CO_2_. To generate SPHK1-knockdown lines, lentiviral particles encoding short hairpin RNAs (shRNAs) targeting three independent SPHK1 sequences (sh1-sh3) or a non-targeting control shRNA were used to infect AGS and HGC27 cells. Transduced cells were selected with puromycin (10 µg/mL) for 6 days to establish stable knockdown pools. Knockdown efficiency was confirmed by Western blotting; two shRNAs, sh1 and sh2, showing the strongest SPHK1 suppression were used for all subsequent experiments. Both control cells expressing non-targeting shRNA and SPHK1-knockdown cells expressing sh1 or sh2 were analyzed in parallel. All assays were performed in both AGS and HGC27 cells, with each condition testes in triplicate, and experiments were repeated at least three times to ensure reproducibility.

### Cell proliferation assay (CCK-8)

Cell proliferation was assessed using a Cell Counting Kit-8 (CCK-8; Dojindo) according to the manufacturer’s protocol. Briefly, control and SPHK1 knockdown-cells were seeded in 96‐well plates at a density of 2 × 10^3^ cells per well. After incubation for 24, 48, or 72 h, 10 µL of CCK-8 reagent was added to each well and the plates were incubated for 2 h at 37 °C. Absorbance at 450 nm was measured using a microplate reader. Cell viability was calculated based on optical density values, and the results are reported as the mean ± SD from three independent experiments.

### Colony formation assay

Cells in the logarithmic growth phase were trypsinized, counted, and diluted into a single-cell suspension. Control and SPHK1-knockdown cells were seeded at low density (100 cells per 6-cm dish) in triplicate and cultured for 10–14 days to allow colony formation. At the endpoint, colonies were rinsed twice with PBS, fixed with 100% methanol (5 mL per dish) for 15 min, and then stained with Giemsa solution for 30 min. Plates were washed with water and air-dried, and visible colonies containing ≥ 10 cells were counted under a light microscope. Colony counts were averaged from triplicate dishes for each condition.

### Wound-healing migration assay

Cell migration was measured using a wound-healing assay. Control and SPHK1-knockdown cells were seeded at 6 × 10^5^ cells per well in 6-well plates and grown to 100% confluence. A straight wound was then created in the cell monolayer using a sterile pipette tip, and detached cells were removed by washing with PBS. Fresh serum-free medium was added, and images of the scratch were captured immediately at 0 h and after 24 h at 4× magnification. All measurements were performed in triplicate wells and averaged across three independent experiments.

### Oxidative stress assay (MDA measurement)

Intracellular oxidative stress was assessed by measuring malondialdehyde (MDA) levels using a commercial lipid peroxidation thiobarbituric acid (TBA) assay kit according to the manufacturer’s instructions. Briefly, cells were lysed, and the lysates were mixed with TBA reaction solution. Samples were heated at 100 °C for 15 min and then cooled on ice. After centrifugation at 1,000×g for 10 min, 200 µL of the supernatant was transferred to a 96-well plate, and absorbance was measured immediately at 532 nm. MDA concentrations were determined by comparison with an MDA standard curve. Each condition was measured in triplicate, and the data are represented as the mean ± SD from three independent assays.

### Alkaline phosphatase (ALP) staining

Cellular differentiation status was evaluated by ALP histochemical staining using BCIP/NBT substrate (Sigma-Aldrich). Cells were fixed with 10% formaldehyde for 15 min at room temperature and washed with PBS. BCIP/NBT solution was then added, and the cells were incubated for 30 min at room temperature. ALP-positive cells developed a dark purple-blue precipitate. Stained cells were imaged under a microscope, and the percentage of ALP-positive cells was calculated. Each staining experiment was performed in both AGS and HGC27 cells in triplicate.

### Western blot analysis

Cells were lysed in RIPA buffer (Beyotime, China) supplemented with protease and phosphatase inhibitors. Equal amounts of protein (30 µg) were separated by 10% SDS-PAGE and transferred onto PVDF membranes (Millipore, United States). Membranes were blocked with 5% non-fat milk in TBST and incubated overnight at 4 °C with primary antibodies against SPHK1 (1:1000; Abcam, ab71700), Bax (1:1000; Cell Signaling Technology, #2772), CDC2 (1:1000; Abcam, ab18), Cyclin D1 (1:2000; Proteintech, 26939-1-AP), p21 (1:1000; Cell Signaling Technology, #2947), p27 (1:1000; Cell Signaling Technology, #3686), TGF-β1 (1:500; R&D Systems, AF-246-NA), N-cadherin (1:5000; Sigma-Aldrich, C3865), E-cadherin (1:5000; Sigma-Aldrich, MAB3199), and α-tubulin (1:5000; Sigma-Aldrich, T6199). After washing, the membranes were incubated with HRP-conjugated secondary antibodies (1:5000; Cell Signaling Technology) for 1 h at room temperature. Signals were visualized using an enhanced chemiluminescence substrate (Thermo) and captured with a Bio-Rad ChemiDoc imaging system.

### Statistical analysis

All quantitative data are presented as the mean ± SD from at least three independent experiments and were analyzed using GraphPad Prism 8. Statistical comparisons between groups were performed using Student’s t-test, with statistical significance defined as *P* < 0.05. Significance levels were denoted as follows: *P < 0.05, **P < 0.01, and ***P < 0.001.

## Results

**Fig. 1:**
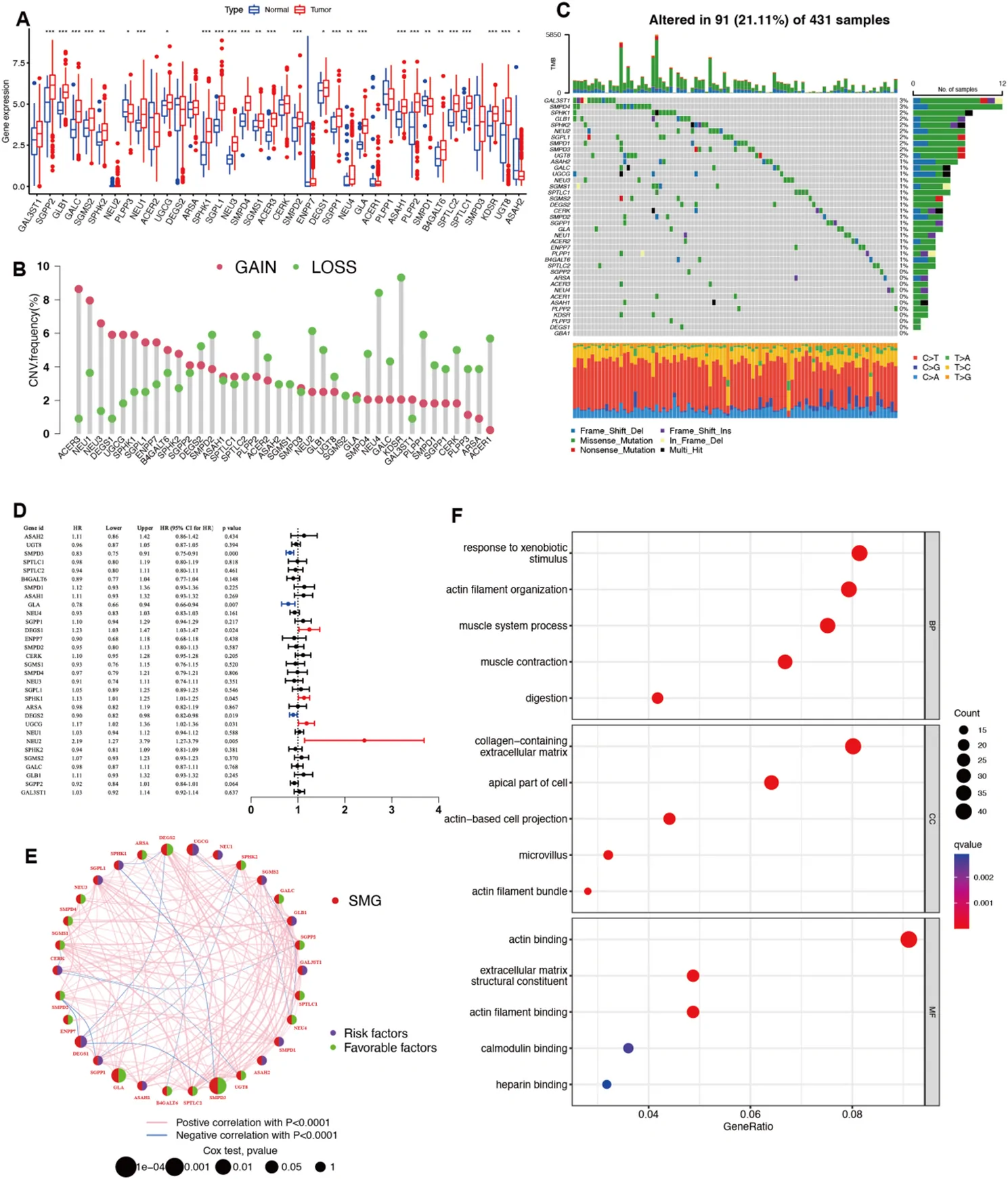
Transcriptional and genomic dysregulation of sphingolipid metabolism genes and their prognostic and functional signatures. **A** Boxplots of expression differences of SMGs in tumor (red) and normal (blue) tissues. Representative genes such as SGPP2, SPHK1, ACER2, CERK are significantly upregulated in tumors, whereas NEU1, SGMS2, B4GALNT1 and others are downregulated, indicating systemic remodeling of sphingolipid metabolism program. **B** Copy number variation (CNV) frequency of SMGs. Grey bars show overall CNV frequency, red dots indicate gains, green dots indicate losses. ACER3 and UGCG are more prone to gains, whereas ASAH2 and SPTLC2 are more frequently deleted. **C** Overview of somatic mutations (oncoplot, TCGA). In total, 91 of 431 (21.1%) samples carry at least 1 SMG alteration. Barplot on right shows mutation frequency for each gene, bar on top shows mutation burden per sample, stacked bars at bottom indicate mutation types. **D** Forest plot of univariate Cox proportional hazards models. Hazard ratio (HR) and 95% confidence interval quantify survival association of each gene. NEU2, DEGS1, CERK and others are adverse prognostic factors (HR > 1), whereas GLA, NEU4 and others are protective factors (HR < 1). **E** SMG coexpression and prognostic network. Nodes represent genes (red = risk factors, green = protective factors; node size proportional to -log10 P value of Cox significance). Edges represent Spearman correlations (red = positive, blue = negative; *P* < 1 × 10^− 4^). Risk module shows strong positive correlations within module and negative connections with protective module. **F** GO enrichment bubble plot stratified by BP, CC, MF. Significant enrichment is observed in processes and structures such as response to xenobiotic stimulus, actin filament organization, muscle system process, collagen containing extracellular matrix, microvillus, and in molecular functions such as actin binding, extracellular matrix structural constituent, calmodulin binding, heparin binding. Horizontal axis shows gene ratio (GeneRatio), dot size indicates number of enriched genes, color indicates adjusted q value. Statistics: panel **A**, Cox correlations, and between group comparisons all use two sided tests; significance indicated as *P < 0.05, **P < 0.01, ***P < 0.001. Abbreviations: SMG, sphingolipid metabolism gene; CNV, copy number variation; HR, hazard ratio; BP/CC/MF, GO biological process, cellular component, molecular function

Sphingolipid metabolism genes (SMGs) exhibited widespread transcriptional dysregulation between tumor and normal tissues (Fig. 1A). Many SMGs were significantly upregulated or downregulated in tumors (*P* < 0.001). For example, SGPP2, SPHK1, GLB1, and PLPP2 were expressed at significantly higher levels in tumor samples, whereas SMPD-1, ASAH2, PLPP3, and others were downregulated. Genomic profiling revealed highly heterogeneous copy number variations (CNVs) among SMGs (Fig. 1B). Genes such as ACER3, NEU1, and NEU3 showed copy number gains in approximately 6–9% of samples, whereas KDSR, NEU4, SMPD2, and others predominantly exhibited copy number losses, with loss frequencies of approximately 6–9%. These findings suggest that CNVs contribute substantially to SMG dysregulation. Although the somatic mutation rate of individual SMGs was generally low, 21.1% of tumor samples harbored multi-gene SMG alterations, including point mutations and CNVs (Fig. 1C), indicating that both types of genetic events jointly contribute to SMG dysregulation. Univariate Cox regression analysis further revealed prognostic heterogeneity among SMGs (Fig. 1D). Notably, NEU2 (HR ≈ 2.19) emerged as a significant marker of poor prognosis, while DEGS1 (HR ≈ 1.23) and SPHK1 (HR ≈ 1.13) were also associated with increased risk. In contrast, GLA (HR ≈ 0.78) and DEGS2 (HR ≈ 0.90) were associated with better outcomes. A network constructed based on SMG expression and co-expression patterns showed that risk-associated genes, such as UGCG and SPHK1 (red nodes in Fig. 1E) interacted extensively with one other, forming a tightly connected module. Conversely, protective genes, such as SMPD3 and GLA (green nodes), such as SMPD3 and GLA (green nodes) negative correlations with each other (Fig. 1E). These findings suggest that distinct functional subsets of SMGs may collectively promote an invasive tumor phenotype through antagonistic regulatory networks. Functional enrichment analysis showed that SMGs were significantly enriched in pathways related to actin filament assembly, muscle system processes, extracellular matrix organization, and cell-matrix adhesion (Fig. 1F). These results highlight the link between sphingolipid metabolic reprogramming, cytoskeletal remodeling, and tumor microenvironment adaptation. Overall, these findings indicate that SMGs undergo coordinated genetic and transcriptional alterations, stratify tumors into subtypes with distinct clinical characteristics, and are closely associated with biological processes that promote tumor progression.

**Fig. 2:**
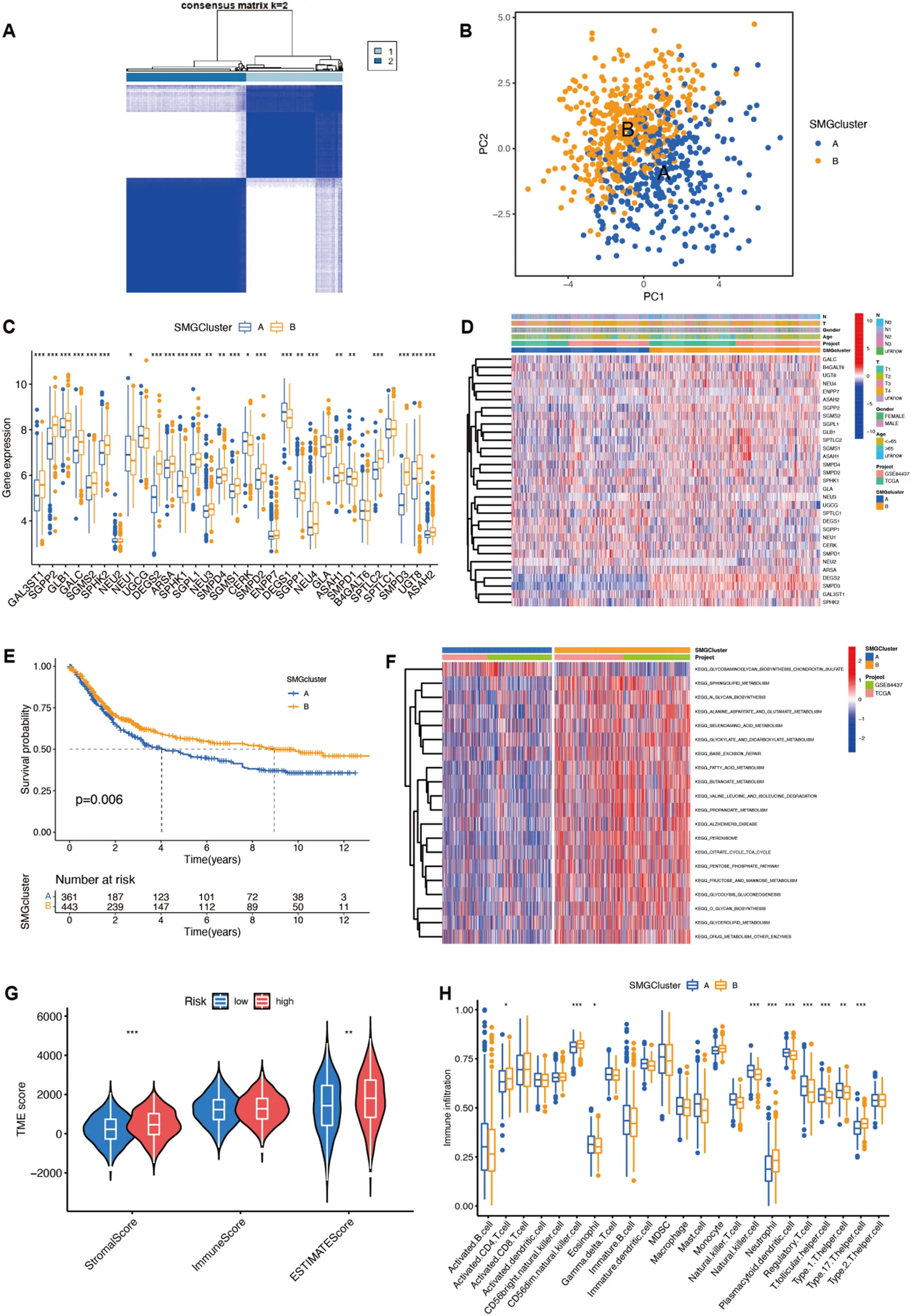
Molecular subtypes based on SMG expression, prognostic differences, and pathway and immune microenvironment features. **A** Consensus clustering heatmap (ConsensusClusterPlus, k = 2). Colors (0–1) represent probability that two samples are assigned to same cluster. Two high concordance blocks along main diagonal define SMGcluster A and SMGcluster B. **B** Principal component analysis (PCA) scatter plot. Separation of two subtypes in PC1-PC2 space based on SMG expression supports robustness of subtyping. **C** Boxplots of gene expression differences between subtypes. Expression of most SMGs is compared between **A** (blue) and **B** (orange/red) using two sided Wilcoxon test. SPHK1 and SGPP2 are higher in **B**, whereas GLA and ACER3/NEU4 are higher in A (*P < 0.05; **P < 0.01; ***P < 0.001). **D** Heatmap of full set. Rows are SMGs, columns are samples (rows and columns both clustered by unsupervised clustering, rows standardized by Z score). Annotation bars on top indicate subtype and clinical features. Two subtypes show stable and coordinated differences at global expression level. **E** Kaplan-Meier overall survival curves. SMGcluster B shows significantly worse prognosis (log rank test *P* < 0.01). Risk table is shown below. **F** Heatmap of pathway level GSVA scores (TCGA and independent GEO datasets). Subtype B is enriched in metabolic pathways such as glycolysis/gluconeogenesis, fatty acid metabolism, pentose phosphate pathway, whereas subtype A is relatively enriched in oxidative phosphorylation and some immune related processes (FDR adjusted *P* < 0.05). **G** Violin plots of tumor microenvironment (TME) scores (ESTIMATE). Compared with A, subtype B shows markedly lower ImmuneScore and ESTIMATEScore, with StromalScore also showing a decreasing trend (two sided Wilcoxon test, significance indicated by asterisks), suggesting an immunosuppressive microenvironment. **H** Immune infiltration comparison (ssGSEA/CIBERSORT). In subtype B, antitumor effector cells such as activated CD8^+^ T cells, dendritic cells, memory B cells are significantly reduced, whereas M2 like macrophages are relatively increased (*P < 0.05), consistent with high-risk characteristics. Statistics and color coding: all panels except **E** use two sided Wilcoxon tests; panel E uses log rank test. Significance is uniformly indicated as *P < 0.05, **P < 0.01, ***P < 0.001. Cluster A is shown in blue and cluster B in orange/red. Abbreviations: GSVA, gene set variation analysis; ESTIMATE/Immune/Stromal/ESTIMATEScore, algorithm and its scores; TME, tumor microenvironment

Building on SMGs associated with tumor progression, we then performed unsupervised consensus clustering (k = 2) using SMG expression. This partitioned the cohort into two robust molecular subtypes: Cluster A and Cluster B (Fig. 2A). The consensus matrix revealed two distinct blocks of high concordance along the diagonal: the co-occurrence probability of samples within the same cluster was near 1, and near 0 between different clusters. This indicates clearly defined subtype boundaries with a minimal probability of cross-cluster misclassification. Principal component analysis using SMG expression similarly supported this separation: Clusters A and B were clearly distinguished in the space defined by the first two principal components (Fig. 2B), indicating that the primary sources of variance arise from SMG program itself rather than from noise.

We then compared the expression levels of individual SMGs between the subtypes. Representative boxplots (Fig. 2C) showed that high-risk Cluster B tended to activate the sphingolipid remodeling program. Sphingosine-1-phosphate phosphatase 2 (SGPP2), delta(4)-desaturase, sphingolipid 2 (DEGS2), and sphingomyelin phosphodiesterase 3 (neutral sphingomyelinase-2, SMPD3) were significantly upregulated in Cluster B (all *P* < 0.001). In contrast, sphingosine kinase 1 (SPHK1), a key enzyme in the S1P production/signaling axis, was enriched in Cluster A (*P* < 0.01). Functionally, SGPP2 catalyzes the dephosphorylation of S1P, SMPD3 promotes the breakdown of sphingomyelin to generate ceramide, and DEGS2 is involved in regulating the degree of ceramide unsaturation. Together, these three genes suggest a metabolic orientation characterized by decreased S1P levels and increased ceramide flux, whereas SPHK1 catalyzes the conversion of sphingosine to S1P and is associated with pro-proliferative and pro-migratory signaling. Collectively, Cluster B is characterized by a molecular profile of enhanced sphingolipid synthesis and turnover, whereas Cluster A is more inclined toward the S1P production/signaling axis.

The global SMG expression heatmap (Fig. 2D) further highlighted these differences. Cluster B tumors exhibited coordinated upregulation of many SMGs (after row normalization, the majority of genes showed higher expression in Cluster B), whereas Cluster A samples tended to exhibit lower expression of these genes. Additionally, the subtype annotations correlated with certain clinical features: for example, the proportion of T4 stage tumors was higher in Cluster A but lower in Cluster B. These findings suggest that the subtypes reflect not only molecular co-expression modules but also correspond to an anatomical progression dimension, with Cluster A displaying more locally advanced TNM stages and Cluster B showing a lower disease burden in terms of tumor invasion. Importantly, Kaplan-Meier survival analysis indicated that patients in Cluster B had significantly longer overall survival than those in Cluster A (log-rank *P* < 0.01; Fig. 2E). Consistently, Cluster A had a higher median risk score based on SMG expression. Collectively, these findings are coherent: the SMG-derived risk signal was more active in Cluster A, consistent with its poorer survival outcome.

Functional enrichment analysis (Fig. 2F) indicated that metabolic pathways, including glycolysis/gluconeogenesis, fatty acid metabolism, and the pentose phosphate pathway were significantly enriched in Cluster B (GSVA adjusted *P* < 0.05), whereas Cluster A was enriched in glycosaminoglycan biosynthesis-chondroitin sulfate. The former suggests metabolic reprogramming that favors energy and precursor supply, redox buffering via the pentose phosphate pathway, and lipid synthesis, whereas the latter points to structural remodeling through glycosaminoglycan metabolism. Together, these findings highlight systematic differences in metabolic networks between the two subtypes. Consistently, ESTIMATE-derived stromal scores and overall ESTIMATE scores were significantly lower in high-risk Cluster A (Fig. 2G; both *P* < 0.001), implying that this subtype has higher tumor purity and a relatively suppressed microenvironment. Correspondingly, immune profiling showed that CD45^+^ natural killer cells, neutrophils, and Th17^+^ helper T cells were significantly increased in Cluster B (*P* < 0.05), whereas plasma cells, follicular helper T cells, and regulatory T cells were relatively elevated in Cluster A (Fig. 2H). The immune context of Cluster B indicates stronger effector immune activity and inflammation, whereas Cluster A reflects a bias toward immunoregulation and humoral immunity. These findings further support a systematic divergence in the immune niche between the two subtypes.

Collectively, evidence across five distinct layers—transcriptional programs, clinical stage, survival outcome, metabolic pathways, and microenvironment composition—forms a consistent chain of evidence that validates the two SMG-defined subtypes and highlights their molecular and clinical phenotypic differences. This firmly links SMG expression patterns with tumor progression and patient prognosis.

**Fig. 3:**
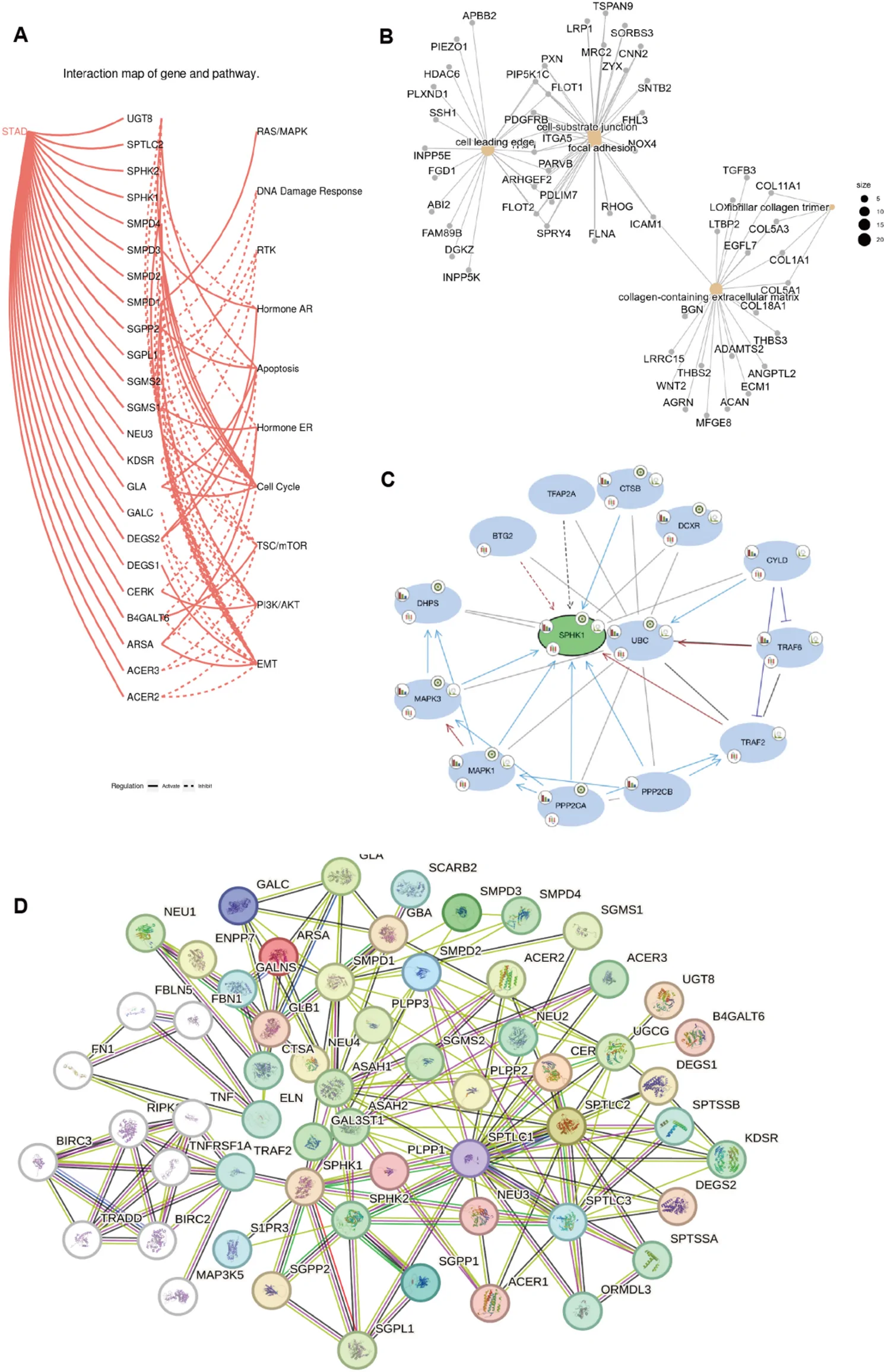
SMG related signaling pathways and interaction networks. **A** Network map of multiple tumor related signaling pathways, showing SMG involvement in core pathways including receptor tyrosine kinase (RTK), PI3K/AKT, MAPK, epithelial mesenchymal transition (EMT), and apoptosis. SMPD3, encoding neutral sphingomyelinase 2, participates in stress signaling and apoptosis process by generating bioactive lipid ceramide. Red lines mark connections between SMPD3 and multiple SMGs, suggesting highly connected hub role in network. **B** SMG-ECM pathway interaction network. Reactome analysis shows that SMGs and genes in extracellular matrix (ECM) related pathways form tight interaction network. **C** Regulatory network centered on key genes. SPHK1 is located at center of network and links PI3K/AKT and MAPK signaling pathways. Upstream and downstream interaction genes shown in plot suggest potential feedback regulatory mechanisms. **D** SMG protein protein interaction network. Network constructed from STRING database shows close interactions among representative SMG genes such as SPHK1, GALC, SGMS2, ACER2, NEU4. These core enzymes act directly or co participate in sphingolipid and glycosphingolipid metabolism pathways and jointly affect cell signal transduction, proliferation, and migration

After characterizing the functional and immune distinctions through SMG-based subtyping, we further constructed and analyzed SMG-associated signaling pathways and interaction networks (Fig. 3). Figure 3A shows that SMGs are involved in several core signaling pathways, including receptor tyrosine kinase (RTK), PI3K/AKT, MAPK, epithelial-mesenchymal transition (EMT), and apoptosis. Among these genes, SMPD3 was connected to multiple SMGs as indicated by the red lines, highlighting its potential role as a highly connected regulatory hub. Reactome pathway analysis (Fig. 3B) revealed that SMGs formed a tightly connected interaction network with genes involved in extracellular matrix (ECM) organization pathways. This suggests that SMGs may influence the tumor microenvironment and invasive capacity by regulating matrix remodeling. A key gene-centered regulatory network (Fig. 3C) further showed that SPHK1 occupied a central position, linking the PI3K/AKT and MAPK signaling pathways. The predicted upstream and downstream interaction partners of SPHK1 suggested potential feedback mechanisms. The protein-protein interaction network of SMGs constructed from STRING database (Fig. 3D) confirmed close connections among representative SMGs; for example, the core genes SPHK1, GALC, SGMS2, ACER2, and NEU4 either directly interacted or jointly participated in sphingolipid/glycosphingolipid metabolic pathways, exerting synergistic effects on signal transduction, cell proliferation, and migration. Taken together, these network analyses highlight the hub roles of SMPD3 and SPHK1 and provide molecular mechanistic evidence for the involvement of SMGs in tumor-related signaling pathways, including RTK, PI3K/AKT, MAPK, EMT, and apoptosis.

**Fig. 4:**
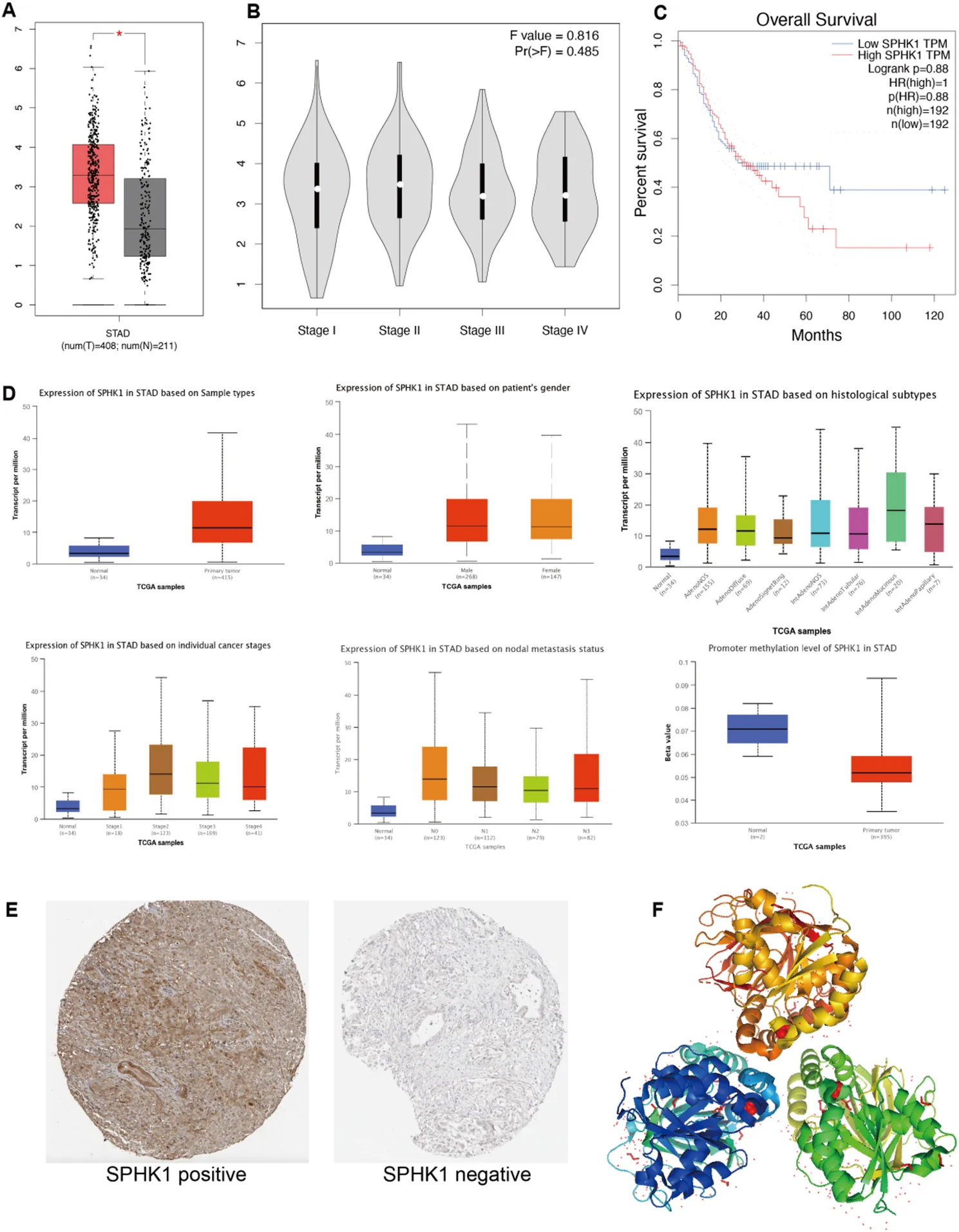
Expression and structural characteristics of SPHK1 in gastric cancer. **A** SPHK1 expression in TCGA STAD cohort. Boxplot comparison shows SPHK1 expression significantly higher in gastric cancer tissues than in normal gastric mucosa group (*P < 0.05). **B** SPHK1 expression across different TNM stages. Boxplots show no significant differences in SPHK1 expression among stage groups (F = 0.816, *P* = 0.485). **C** Overall survival analysis. Kaplan-Meier curves compare SPHK1 high expression group and low expression group; overall survival shows no significant difference (log rank *P* = 0.88). **D** Clinical subtype comparison. Heterogeneity of SPHK1 expression across groups defined by sex, histological subtype, pathological stage, metastatic status, and promoter methylation level of SPHK1. **E** Immunohistochemical staining. SPHK1 shows strong positive staining (dark brown) in gastric cancer tissues, whereas only weak staining is observed in normal gastric mucosa. **F** SPHK1 protein structure. X ray crystal structure of human SPHK1 protein monomer (PDB ID 3VZB)

Considering gene network centrality, consistency with expression-based subtyping, and experimental feasibility, we prioritized SPHK1 for functional validation in subsequent studies. Analysis of TCGA data showed that SPHK1 expression was significantly higher in gastric cancer tissues than in normal gastric mucosa (*P* < 0.05; Fig. 4A), consistent with its high expression in Cluster A among our SMG-defined subtypes. By contrast, SPHK1 levels did not differ significantly across TNM stage groups (F = 0.816, *P* = 0.485; Fig. 4B), suggesting that its expression does not markedly change with disease progression. Kaplan-Meier survival analysis indicated that the high-SPHK1-expression group had a slightly lower overall survival rate than the low-expression group, although the difference was not statistically significant (log-rank *P* = 0.88; Fig. 4C). This trend is consistent with the biological pattern observed in the high-risk SMG subtype, Cluster A, in which SPHK1 is upregulated, although the lack of statistical significance is likely attributable to limitations in sample size or effect size. Boxplots of clinical subgroups (Fig. 4D) further revealed slightly higher SPHK1 expression in male patients, although this difference was not statistically significant. SPHK1 levels also exhibited heterogeneity across histological subtypes, pathological stages, and metastatic statuses, such as among tubular adenocarcinoma cases. Moreover, although SPHK1 expression was markedly higher in tumors than in normal tissues, its promoter methylation level was significantly lower, suggesting a potential epigenetic de-repression mechanism. Immunohistochemical staining (Fig. 4E) confirmed the transcriptomic findings: SPHK1 displayed strong positive expression in gastric cancer tissue, whereas only weak staining was observed in normal gastric mucosa. In addition, Fig. 4F shows the X-ray crystallography-resolved three-dimensional structure of the SPHK1 protein (PDB ID: 3VZB), which currently represents the most accurate model of the SPHK1 monomer. In particular, chain C, in the ligand-free state, most closely resembles the monomeric conformation that performs the catalytic function under physiological conditions. The structure reveals a conserved overall fold and the spatial arrangement of key catalytic domains, such as the dinucleotide-binding site, providing structural insights to support subsequent functional studies.

**Fig. 5:**
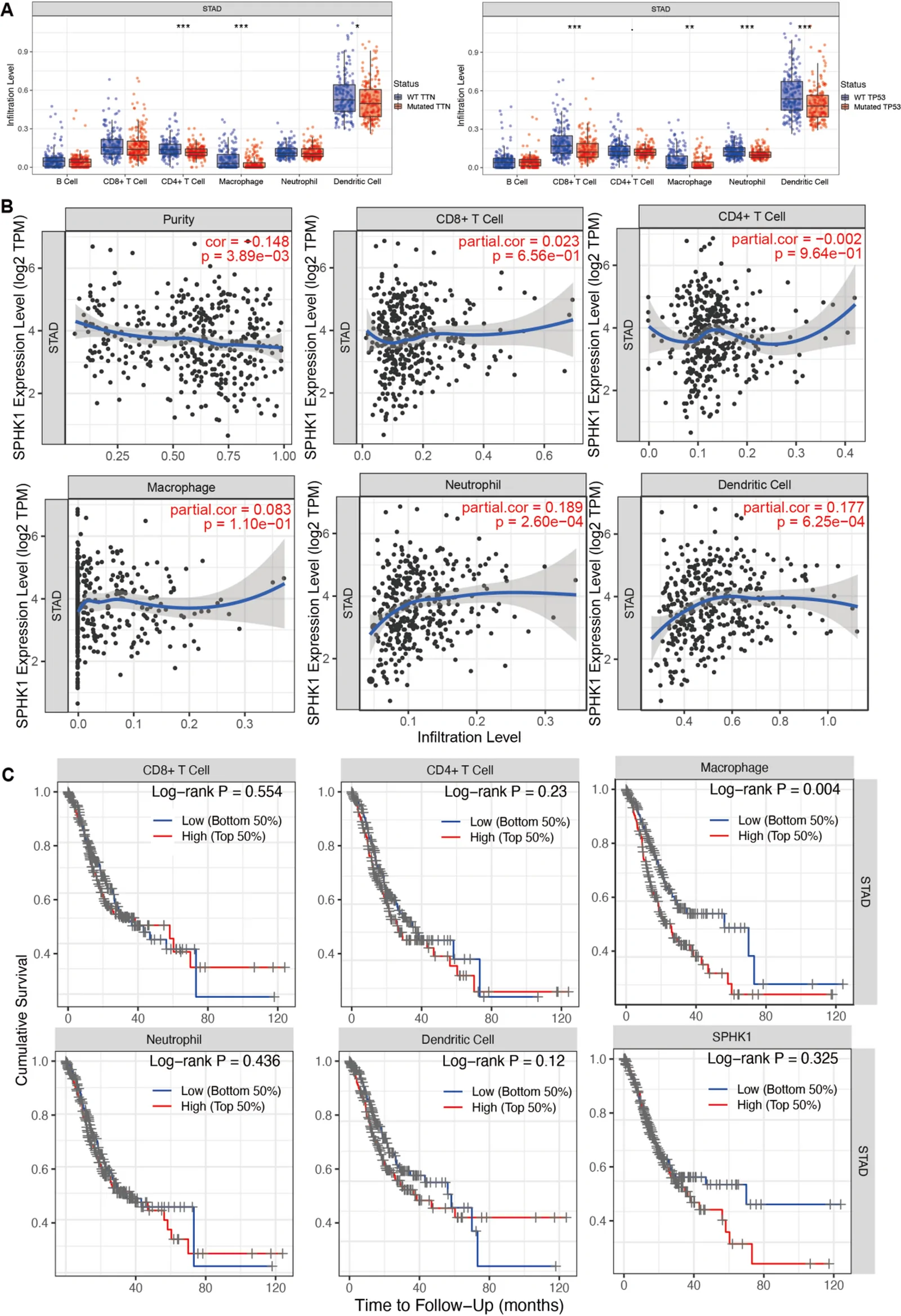
Analysis of association between SPHK1 and tumor immune infiltration. **A** Driver mutations and immune infiltration. In SPHK1 high expression tumor samples, TTN mutant subgroup shows inflamed immune microenvironment, whereas TP53 mutant subgroup shows relatively suppressive features. **B** TIMER correlation analysis. SPHK1 expression shows positive correlation with tumor purity (*r* = 0.148, *P* = 3.89 × 10^− 3^). SPHK1 shows significant positive correlation with neutrophil (partial *r* = 0.189, *P* = 2.60 × 10^− 4^) and dendritic cell (partial *r* = 0.177, *P* = 6.25 × 10^− 4^) infiltration; correlation with macrophages is weaker (partial *r* = 0.083, *P* = 0.11); correlations with CD8^+^ T cells (*r* = 0.023, *P* = 0.066) and CD4^+^ T cells (*r* = −0.002, *P* = 0.964) are not significant. **C** Immune stratified survival. Kaplan-Meier analysis in high immune infiltration subgroups. Only in macrophage high infiltration group do patients with high SPHK1 expression show significantly worse overall survival (log rank *P* = 0.004). In CD8^+^/CD4^+^ T cell, neutrophil, and dendritic cell high infiltration groups, survival differences between high and low expression groups are not significant (*P* > 0.1). Survival difference in whole cohort dichotomized by SPHK1 expression is also not significant (*P* = 0.325)

To further investigate immunological significance of SPHK1 (Fig. 5), we performed a stratified analysis of tumor immune microenvironment (TIME) and integrated these data with common driver mutations. Within a framework combining immune infiltration and gene mutations (Fig. 5A), under conditions of high SPHK1 expression, samples with TTN mutations exhibited a more inflamed TIME, as evidenced by markedly increased infiltration of CD4^+^ T cells, dendritic cells (DCs), and macrophages. In contrast, samples harboring TP53 mutations displayed an immune-suppressive phenotype: characterized by overall reduced infiltration of CD8^+^ T cells, macrophages, neutrophils, and DCs, along with enhanced immunosuppressive signatures. These results indicate that different driver mutations may be associated with distinct SPHK1-related immune contexts. Further correlation analysis using TIMER (Fig. 5B) showed that SPHK1 expression was negatively correlated with tumor purity (cor = −0.148, *P* = 3.89 × 10^− 3^). After controlling for tumor purity, SPHK1 expression remained significantly positively correlated with neutrophil infiltration (partial cor = 0.189, *P* = 2.60 × 10^− 4^) and dendritic cell infiltration (partial cor = 0.177, *P* = 6.25 × 10^− 4^). Its correlation with macrophages was weaker (partial cor = 0.083, *P* = 0.11), and correlations with CD8^+^ T cells (partial cor = 0.023, *P* = 0.066) and CD4^+^ T cells (partial cor = −0.002, *P* = 0.964) were not significant. These data suggest that high SPHK1 expression is more likely accompanied by an enrichment of myeloid and antigen-presenting cells, particularly neutrophils and DCs, with relatively little direct association with effector T cell infiltration. Survival stratification further confirmed its clinical relevance (Fig. 5C). Only in the subgroup with high macrophage infiltration did high SPHK1 expression predict significantly worse overall survival (log‑rank *P* = 0.004). In subgroups with high CD8^+^ T cell, CD4^+^ T cell, neutrophil, or DC infiltration, survival differences between high and low SPHK1 expression were not significant (all *P* > 0.1). In the overall cohort (without immune stratification), the survival differences by SPHK1 expression was also not significant (*P* = 0.325). Taken together, SPHK1 expression is consistently positively associated with myeloid cell (neutrophil/DC) infiltration and is linked to worse outcomes in a macrophage-rich immune context. This immune landscape mirrors immunosuppressive characteristics of SMG Cluster A described above, suggesting an connection between high SPHK1 expression and a myeloid-enriched immunosuppressive microenvironment, particularly in relation to tumor-associated macrophages. These findings provide a clear rationale for subsequent functional experiments and potential therapeutic interventions targeting SPHK1.

**Fig. 6:**
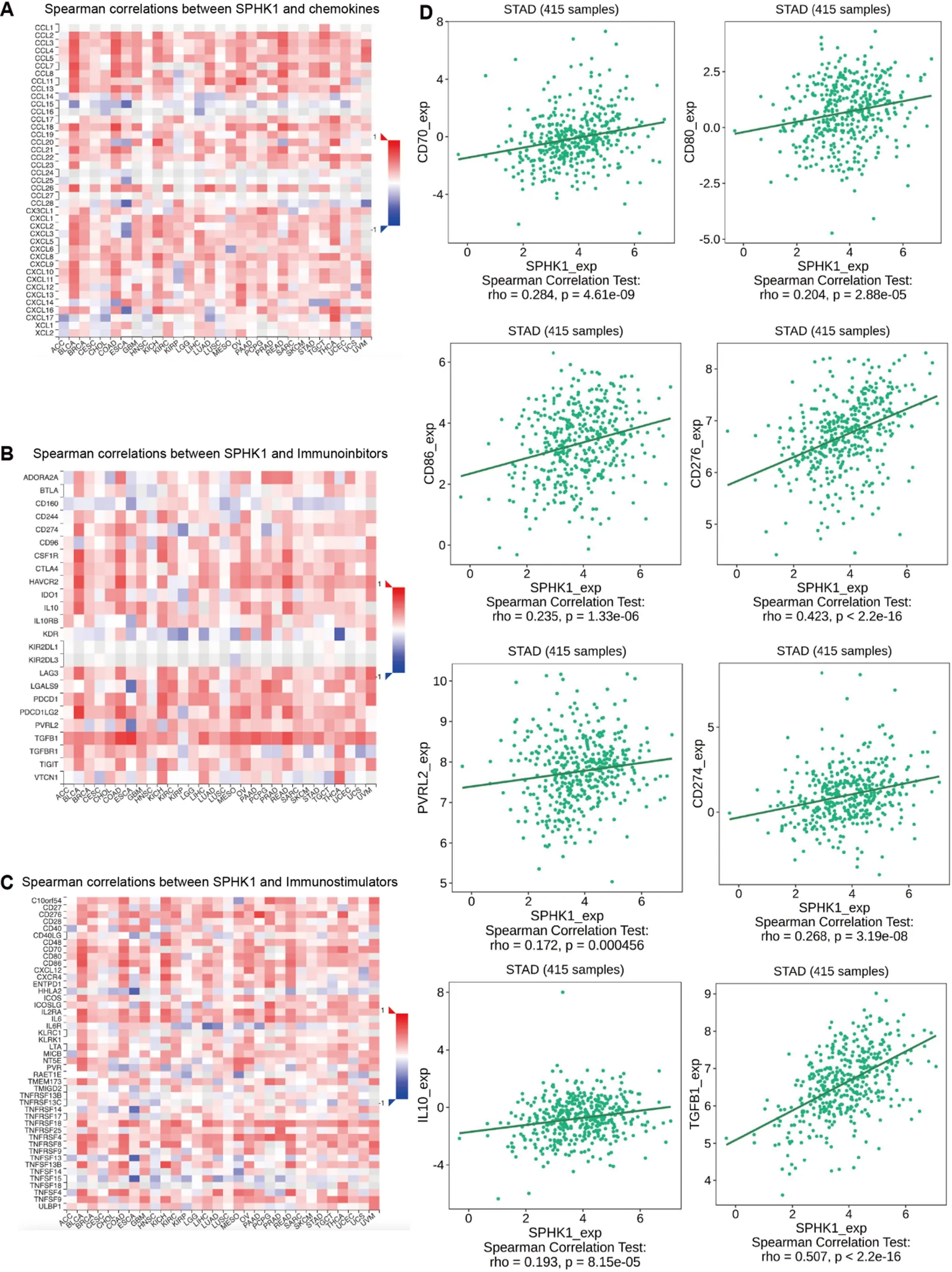
Pan cancer correlations between SPHK1 and immune related molecules and validation in STAD. **A** Heatmap of chemokine correlations from TISIDB database. Spearman correlation coefficients between SPHK1 and multiple chemokines across cancer types (red = positive correlation, blue = negative correlation). Representative molecules include CCL2, CCL5, CXCL8, CCL20, CXCL12, CSF1, CSF2, XCL1 and others. **B** Heatmap of correlations with immunosuppressive factors. SPHK1 shows widespread positive correlations with TGFB1, CD276 (B7‑H3), CD274 (PD‑L1), PDCD1LG2 (PD‑L2), HAVCR2 (TIM‑3), LAG3, CTLA4 and others across cancer types. **C** Heatmap of correlations with immunostimulatory factors. SPHK1 shows predominantly positive correlations with CD70, CD80, CD86, CD40, ICOSLG, members of TNFRSF family. **D** Validation scatter plots in STAD cohort (*n* = 415). SPHK1 shows significant positive correlations with CD70 (*r* = 0.284, *P* = 4.61 × 10^− 9^), CD80 (*r* = 0.204, *P* = 2.88 × 10^− 5^), CD86 (*r* = 0.235, *P* = 1.33 × 10^− 6^); also shows significant positive correlations with CD276/B7‑H3 (*r* = 0.423, *P* < 2.2 × 10^− 16^), CD274/PD‑L1 (*r* = 0.268, *P* = 3.19 × 10^− 8^), PVRL2/CD112 (*r* = 0.172, *P* = 4.56 × 10^− 4^), IL‑10 (*r* = 0.193, *P* = 8.15 × 10^− 5^), and TGF‑β1 (*r* = 0.507, *P* < 2.2 × 10^− 16^). Data source: TISIDB

Building on the immune findings described above, we next used TISIDB platform to map the associations between SPHK1 and various immune-related molecules across cancers (Fig. 6). At chemokine level (Fig. 6A), SPHK1 exhibited broadly positive correlations with numerous chemokines involved in recruiting myeloid cells and T cells. Representative examples include CCL2/CCL5 (which recruit monocytes and memory T cells), CXCL8 (recruits neutrophils), CCL20 (recruits DCs and Th17 cells), and CXCL12, among others. Across many cancer types, the correlation heatmap is predominantly red, indicating that SPHK1 upregulation is accompanied by enhanced immune cell trafficking signals. Regarding immunosuppressive molecules (Fig. 6B), SPHK1 was positively correlated with TGFB1, CD276 (B7‑H3), PDCD1LG2 (PD‑L2), HAVCR2 (TIM‑3), LAG3, CTLA4, and other checkpoint or inhibitory axis components, suggesting a tendency toward co-activation of immunoinhibitory pathways. Conversely, immunostimulatory molecules (Fig. 6C) such as CD70, CD80, CD86, and members of TNFRSF family also exhibited generally positive correlations with SPHK1, indicating that, alongside inflammatory recruitment signals, antigen presentation and co-stimulatory pathways are simultaneously upregulated. This generates an immune state of concurrent activation and inhibition. Focusing on the STAD cohort (Fig. 6D), scatter plots further quantified these associations: CD70 (*r* = 0.284, *P* = 4.61 × 10^− 9^), CD80 (*r* = 0.204, *P* = 2.88 × 10^− 5^), and CD86 (*r* = 0.235, *P* = 1.33 × 10^− 6^) each showed significant positive correlations with SPHK1. Among inhibitory molecules, CD276 (B7-H3) had the strongest correlation (*r* = 0.423, *P* < 2.2 × 10^− 16^), and TGFB1 was even more pronounced (*r* = 0.507, *P* < 2.2 × 10^− 16^). CD274 (PD‑L1) and PVRL2 (CD112) also showed moderate positive correlations with SPHK1 (*r* = 0.268, *P* = 3.19 × 10^− 8^; *r* = 0.172, *P* = 4.56 × 10^− 4^, respectively). IL10 was likewise positively correlated (*r* = 0.193, *P* = 8.15 × 10^− 5^). Integrating the heatmap and STAD scatter results, SPHK1 is positively associated with both chemokine/co-stimulatory and checkpoint/immunosuppressive axes, illustrating an immune landscape in which heightened inflammatory recruitment is counterbalanced by inhibitory networks. This scenario is consistent with the findings in Fig. 5, where SPHK1 correlates with neutrophil/DC infiltration yet is linked to worse prognosis in an immunosuppressive context, and it echoes the immunosuppressive nature of SMG Cluster A. Collectively, these results indicate that SPHK1 may contribute to a inflamed-but-ineffective tumor immune microenvironment in gastric cancer by coupling chemotactic myeloid recruitment with upregulation of inhibitory pathways including TGF-β, B7-H3, and PD-L1.

**Fig. 7:**
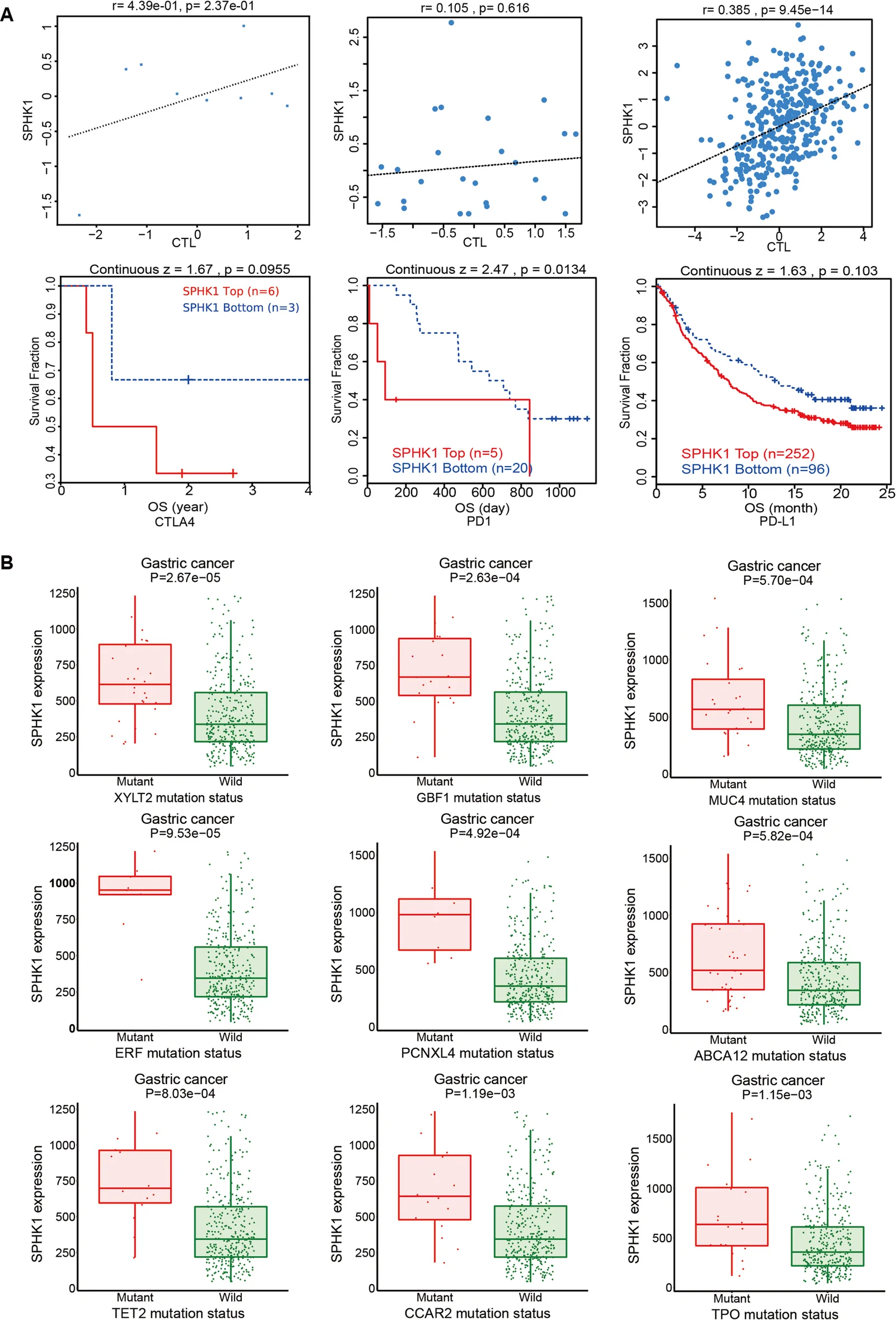
Association of SPHK1 with immune checkpoints and mutational background. **A** Relationship between SPHK1 and immune checkpoint molecule expression and overall survival in STAD cohort. Scatter plot shows correlation between SPHK1 and PD-1 (*r* = 0.385, *P* = 9.45 × 10^− 14^). Within SPHK1 high expression population, PD-1 high expression subgroup has shorter overall survival (Cox model with continuous variable, Z = 2.47, *P* = 0.0134). Combined survival effects of SPHK1 with CTLA4 and PD‑L1 do not reach significance (Z = 1.67, *P* = 0.0955; Z = 1.63, *P* = 0.103). **B** Mutation stratified expression analysis based on muTarget (TCGA STAD). Boxplots compare SPHK1 expression between mutant and wild-type groups for genes XYLT2, GBF1, MUC4, ERF, PCNXL4, ABCA12, TET2, CCAR2, TPO; all mutant groups show significantly higher SPHK1 expression than wild-type (two sided tests, all *P* < 0.01)

To construct a continuous evidence chain with the immunologic results described above, we further evaluated the relationship between SPHK1 and immune checkpoint pathways as well as genomic context (Fig. 7). Figure 7A shows that SPHK1 expression was significantly positively correlated with PD-1 expression (*r* = 0.385, *P* = 9.45 × 10^− 14)^. Among patients with high SPHK1 expression, the subgroup with high PD-1 expression exhibited markedly shorter overall survival (Z = 2.47, *P* = 0.0134). In contrast, the combined effects of SPHK1 with CTLA4 or PD-L1 did not reach statistical significance (Z = 1.67, *P* = 0.0955; Z = 1.63, *P* = 0.103, respectively). These observations are consistent with the previously described TIME landscape (“enhanced myeloid recruitment plus upregulated inhibitory axes” in Figs. 5 and 6), suggesting that PD-1 pathway may represent the primary axis through which SPHK1 mediates immune evasion. Next, Fig. 7B presents an analysis based on the muTarget platform on TCGA STAD data. In samples harboring mutations in any of nine genes (including XYLT2, GBF1, MUC4, ERF, PCNXL4, ABCA12, TET2, CCAR2, and TPO), SPHK1 expression was significantly higher than in the corresponding wild-type samples (all *P* < 0.01). This suggests that these mutational backgrounds are associated with an activated SPHK1 state and may represent upstream drivers or co-occurring events. Taken together, Fig. 7 links the prognostic implication of SPHK1 via PD-1 axis with its elevated expression across specific mutation contexts. These findings further reinforce SPHK1 as a key node in TIME reprogramming and a potential biomarker for immunotherapy response.

**Fig. 8:**
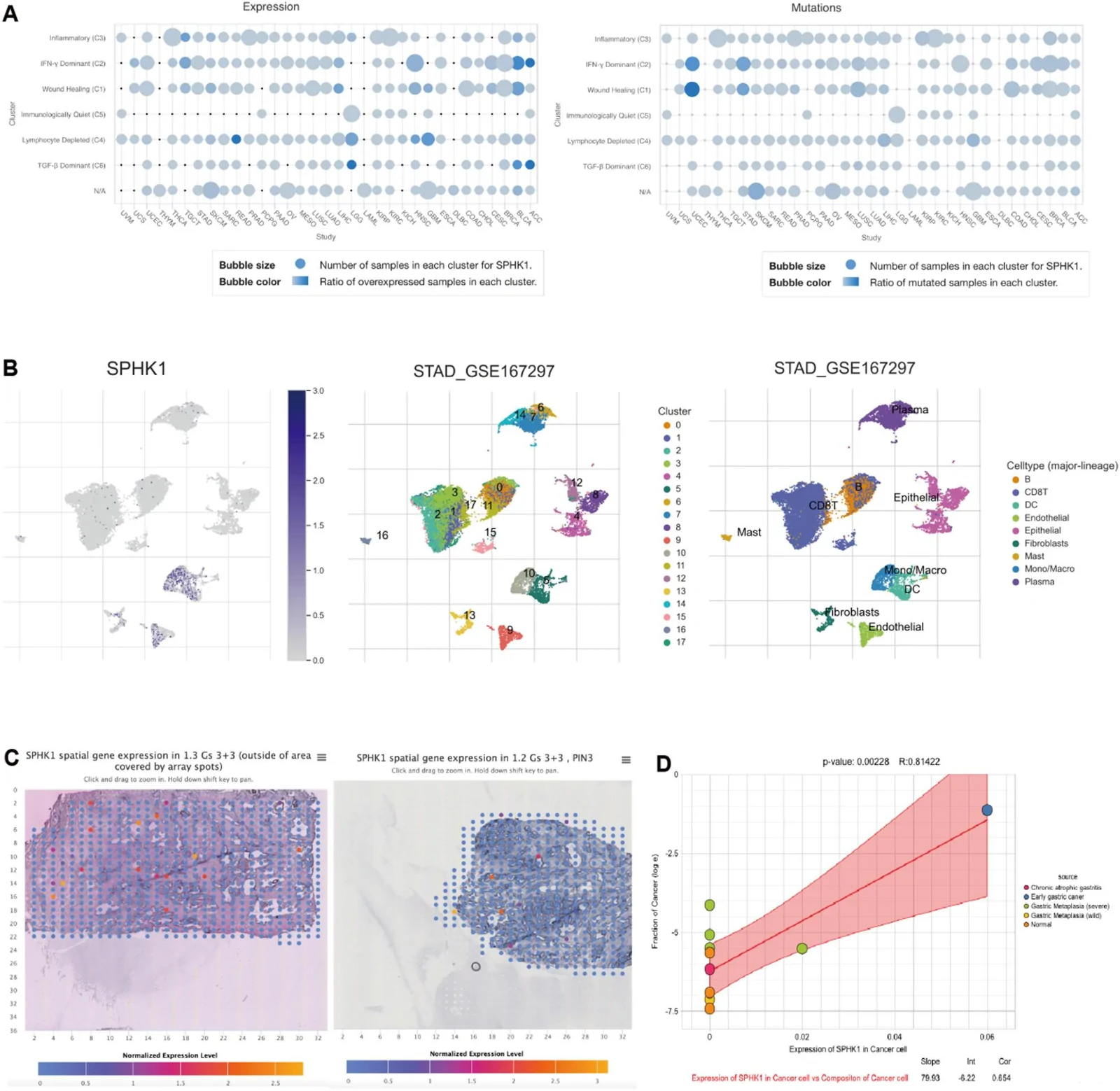
Distribution patterns of SPHK1 in tumor immune subtypes, gastric cancer single cell atlas, and spatial transcriptomics. **A** SPHK1 expression and mutation distribution across immune subtypes. Analysis based on six tumor immune subtypes (C1-C6) constructed by Thorsson, comparing SPHK1 RNA expression and mutation frequency in each subtype. **B** Single cell transcriptome analysis of gastric cancer in GSE167297 dataset. Localization of SPHK1 in cell subpopulations annotated by Seurat. **C** Spatial transcriptomics from SpatialDB. Intratissue spatial enrichment of SPHK1 in prostate tissue (Gleason 3 + 3 region). SPHK1 expression shows focal enhancement in tissue section, with obvious SPHK1 enriched hotspots in tumor areas, whereas adjacent stromal or normal areas show almost no SPHK1 expression. **D** Correlation with scTIME immune evasion scores. SPHK1 expression level shows significant positive correlation with immune evasion score

We then performed an integrated analysis of the distribution and function of SPHK1 within the tumor immune microenvironment. At the bulk immunogenomic level (Fig. 8A), we compared SPHK1 RNA expression and mutation frequency across distinct immune contexts using the six immune subtypes defined by Thorsson et al. in TCGA: C1 wound healing, C2 IFN-γ dominant, C3 inflammatory, C4 lymphocyte-depleted, C5 immunologically quiet, and C6 TGF-β dominant. SPHK1 expression was higher in the C3 inflammatory subtype, whereas its mutation frequency was higher in the C6 TGF-β dominant subtype. These findings indicate that SPHK1 exhibits high expression in an inflammation-rich environment and a higher frequency of genomic alteration in a TGF-β driven suppressive environment, according to the TCGA Immunity 2018 classification. At single-cell resolution (Fig. 8B), analysis of a gastric cancer single-cell RNA-seq dataset (GSE167297, comprising 23,060 cells annotated by Seurat) showed that SPHK1 was mainly expressed by epithelial/tumor cells, with extremely low expression in T cells, mast cells, and stromal cells. Moreover, within the tumor epithelial compartment, a high-SPHK1 subcluster was identified, revealing intratumoral heterogeneity in SPHK1 expression. At the spatial level (Fig. 8C), analysis of the SpatialDB spatial transcriptomic atlas of human prostate cancer tissue, specifically a Gleason 3 + 3 low-grade region, showed that SPHK1 exhibited focal hotspots within tumor areas and scarce expression in adjacent normal/stromal areas. This pattern suggests that SPHK1 may mark a local immune-cold niche within the tissue; however, the prostate sample was used here to illustrate a spatial enrichment pattern and is not directly analogous to gastric cancer. At the functional level (Fig. 8D), using a scTIME derived single-cell immune evasion score, SPHK1 expression was positively correlated with the evasion score (Spearman *P* < 0.001), indicating that higher SPHK1 expression corresponds to a stronger tendency toward immune evasion. Viewed across these complementary scales, these results form a cell-to-tissue-to-space-to-function evidentiary loop demonstrating the involvement of SPHK1 in immune suppression and immune evasion, thereby providing a solid foundation for subsequent experimental validation.

**Fig. 9:**
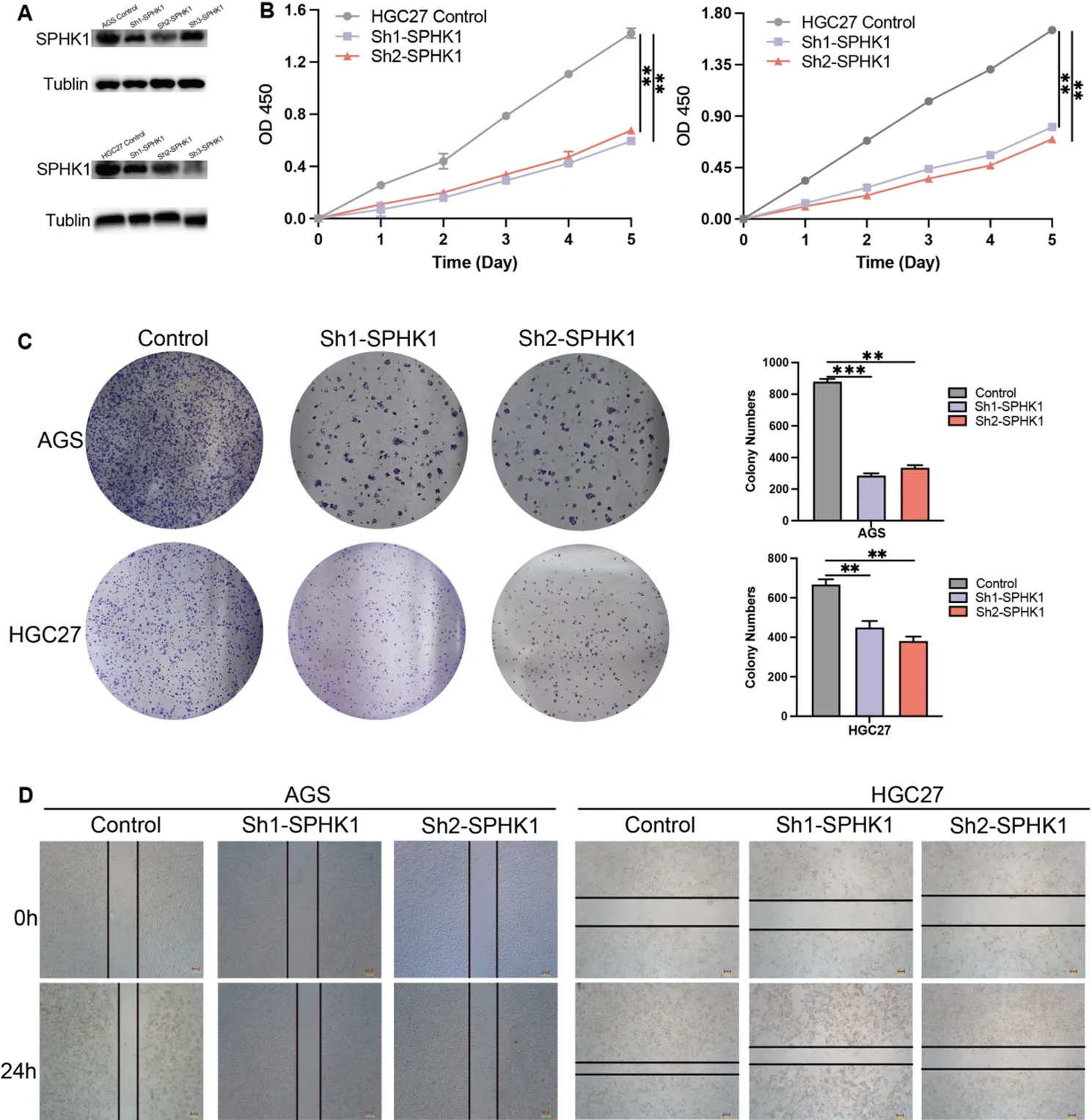
SPHK1 knockdown suppresses proliferation and migration of gastric cancer cells. **A** Western blot validation of SPHK1 knockdown efficiency in AGS and HGC27 cells; control is non targeting shRNA (Control), Tubulin used as internal reference. Two independent shRNAs (sh1‑SPHK1, sh2‑SPHK1) both markedly reduce SPHK1 protein level. **B** CCK‑8 proliferation curves over time (OD_450_, 0–5 days). Compared with control, SPHK1 knockdown significantly reduces proliferation rate of both cell lines. **C** Colony formation assay and quantification. Representative crystal violet staining images (left) and colony number statistics (right). SPHK1 knockdown significantly reduces colony forming ability of AGS and HGC27 cells. **D** Wound healing assay. Representative images at 0 h and 24 h and comparison of wound closure. SPHK1 knockdown markedly delays wound closure, indicating reduced migration capacity. Statistics: data are presented as mean ± s.d.; significance indicated in plots (*P < 0.05, **P < 0.01, ***P < 0.001, two sided tests; specific methods described in Statistical analysis)

Given the high expression of SPHK1 in tumor epithelial cells, its focal spatial enrichment, and its association with immune evasion (Fig. 8), we subsequently knocked down SPHK1 in the gastric cancer cell lines AGS and HGC27 to systematically evaluate its cellular effects (Fig. 9). As shown in Fig. 9A, Western blot analysis first confirmed efficient SPHK1 knockdown in both AGS and HGC27 cells. In the sh1-SPHK1 and sh2-SPHK1 groups, SPHK1 protein expression was markedly reduced, indicating successful knockdown. The CCK-8 proliferation assay (Fig. 9B) demonstrated that SPHK1 knockdown significantly suppressed the proliferative activity of both cell lines; over time, the increase in OD values was noticeably slower in the sh-SPHK1 groups. Consistently, colony formation assays (Fig. 9C) showed that SPHK1 knockdown led to a significant reduction in the number of colonies formed by AGS and HGC27 cells, suggesting that SPHK1 supports the long-term proliferative and clonogenic capacity of tumor cells. Wound-healing scratch assays (Fig. 9D) indicated that SPHK1 knockdown significantly delayed cell migration, as evidenced by a markedly smaller wound closure area in the knockdown groups than in the control groups after 24 h. The functional validation experiments in Fig. 9 therefore demonstrate that SPHK1 inhibition significantly weakens the proliferative and migratory abilities of gastric cancer cells. These in vitro results are consistent with previous studies and highlight the critical role of SPHK1 as a pro-tumorigenic factor in gastric cancer.

**Fig. 10:**
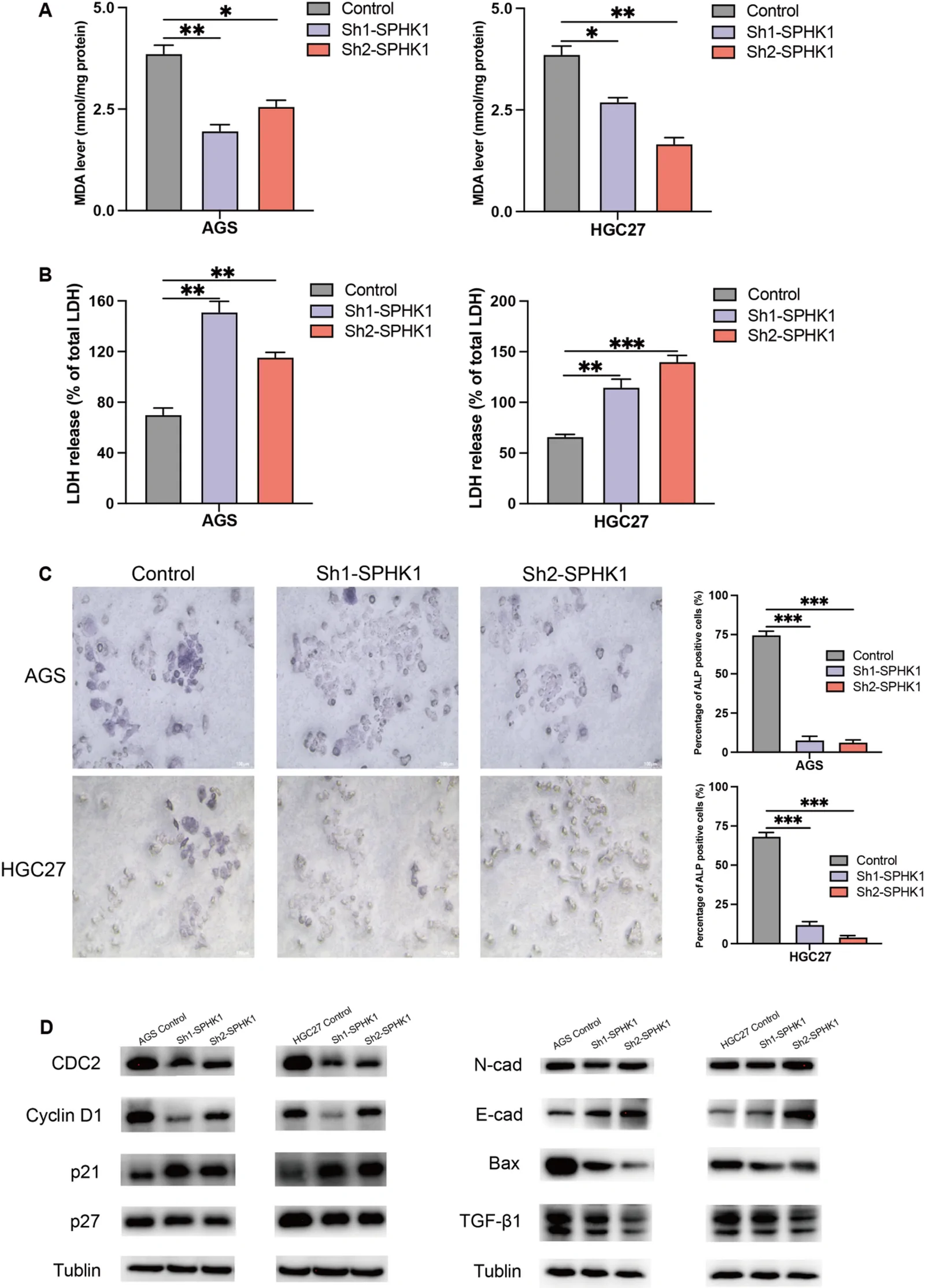
SPHK1 knockdown remodels oxidative stress, cytotoxicity, and molecular phenotype. **A** MDA content (nmol/mg protein) measuring lipid peroxidation level. SPHK1 knockdown significantly reduces MDA levels in AGS and HGC27 cells. **B** LDH release (percentage of total LDH) reflecting cell membrane permeability and cytotoxicity. SPHK1 knockdown significantly increases LDH release. **C** ALP staining and quantification. Proportion of ALP positive cells increases markedly after SPHK1 knockdown, indicating enhanced differentiation tendency and consistent with EMT inhibition. **D** Western blot analysis of key molecules. Cell cycle related CDC2 and Cyclin D1 are downregulated, p21 and p27 are upregulated; EMT related N‑cadherin is downregulated and E‑cadherin is upregulated; pro apoptotic Bax is upregulated; immune and suppressive axis factor TGF‑β1 is downregulated; Tubulin used as internal reference. Statistics: data are presented as mean ± s.d.; significance indicated in plots (*P < 0.05, **P < 0.01, ***P < 0.001, two sided tests; specific methods described in Statistical analysis)

As shown in Fig. 10A, SPHK1 knockdown significantly decreased malondialdehyde (MDA) levels in AGS and HGC27 cells, suggesting reduced lipid peroxidation and attenuated oxidative stress. Figure 10B shows that lactate dehydrogenase (LDH) release into the culture supernatant was significantly increased in SPHK1-knockdown cells, indicating greater cell membrane permeability damage and enhanced cytotoxicity. In Fig. 10C, alkaline phosphatase (ALP) staining revealed that the proportion of ALP-positive cells was markedly increased after SPHK1 knockdown, suggesting an increased tendency toward differentiation, which may be related to regulation of the epithelial-mesenchymal transition (EMT). Figure 10D presents Western blot results showing that SPHK1 silencing led to downregulation of the cell cycle proteins CDC2 and Cyclin D1 and upregulation of the cell cycle inhibitors p21 and p27, indicative of cell cycle arrest. Regarding EMT markers, N-cadherin was downregulated, whereas E-cadherin was upregulated, indicating that the EMT process was inhibited. The pro-apoptotic protein Bax was upregulated, suggesting enhanced apoptotic signaling. In addition, the expression of the immunoregulatory factor TGF-β1 was reduced, suggesting that SPHK1 inhibition may be associated with decreased TGF-β1-related signaling activity; however, further validation using downstream pathway markers, such as p-SMAD2/3, is needed. These results indicate that SPHK1 knockdown suppresses the malignant phenotype of gastric cancer cells by reducing oxidative stress, increasing cytotoxicity, and promoting differentiation. Concomitantly, SPHK1 knockdown induces cell cycle arrest, inhibits EMT, activates apoptotic responses, and diminishes immunosuppressive pathway activity.

## Discussion

Our study demonstrates that sphingolipid metabolism-related genes (SMGs) exhibit widespread transcriptional dysregulation in gastric cancer tissues, which, together with genomic alterations, may contribute to tumor progression [43–45]. Many SMGs were significantly upregulated or downregulated in tumor tissue, reflecting dysregulation of the sphingosine-ceramide-S1P metabolic axis [46, 47]. This observation is consistent with previous studies showing that sphingolipid metabolites regulate cell-cycle proteins, such as Cyclin D1/CDK4, and transcription factors involved in cell proliferation, and promote cell migration and invasion by remodeling the extracellular matrix (ECM) and modulating cell adhesion molecules, for example, through the induction of matrix metalloproteinases [48–52]. In addition, genomic profiling revealed substantial CNV heterogeneity among SMGs, suggesting that these genetic events may drive dysregulation of sphingolipid metabolism [53, 54].

A key contribution of our study is not only that it confirms the dysregulation of SMGs in gastric cancer but also that it integrates transcriptional and genomic features to demonstrate a close association between sphingolipid metabolic imbalance and tumor progression. Rather than supporting a view of SMGs as isolated molecular events, our findings indicate that they function as a coordinated regulatory network that may influence proliferation, survival, invasion, and disease aggressiveness. This integrative perspective provides a stronger biological basis for considering sphingolipid metabolism as a clinically relevant dimension of gastric cancer.

A particularly novel aspect of this study is that unsupervised clustering based on SMG expression identified two molecular subtypes of gastric cancer, Cluster A and Cluster B, with distinct biological behaviors and clinical outcomes [55, 56]. This finding extends beyond descriptive molecular classification by linking sphingolipid metabolism to clinically meaningful differences in prognosis and tumor microenvironment status. Cluster A, the high-risk subtype, was characterized by enrichment of pathways related to glycosaminoglycan synthesis and EMT, lower stromal scores, higher tumor purity, and an immune contexture suggestive of relative immunosuppression, including increased regulatory T-cell infiltration and reduced effector lymphocyte infiltration [57–60]. In contrast, Cluster B, the low-risk subtype, was enriched in glycolysis/gluconeogenesis, fatty acid metabolism, and pentose phosphate pathway activity and showed increased infiltration of natural killer cells, Th17 cells, and neutrophils, indicating a more inflammatory and immune-infiltrated phenotype [61–63].

These observations have important clinical implications. First, the SMG-based clustering framework may help refine risk stratification in gastric cancer by identifying subgroups with different survival probabilities and distinct tumor ecological states. Second, the two subtypes may display different therapeutic vulnerabilities: Cluster A appears to represent a structurally remodeled and immunosuppressive phenotype that may require strategies targeting stromal barriers, EMT-related programs, or immune suppression, whereas Cluster B may be more responsive to approaches that exploit metabolic dependency and pre-existing immune activation [64–68]. In this context, our unsupervised clustering analysis provides a molecularly informed basis for patient stratification and highlights the translational potential of sphingolipid metabolism as both a prognostic indicator and a potential guide for personalized therapeutic decision-making.

Within the SMG network, SPHK1 emerged as a key hub gene. SPHK1 encodes sphingosine kinase 1, which catalyzes the conversion of ceramide/sphingosine to S1P [69]. SPHK1 has been reported to be overexpressed in multiple cancers and associated with poor prognosis [30]. Our analysis showed that SPHK1 was significantly upregulated in gastric cancer tissues, consistent with the high-risk subtype, and this was accompanied by a trend toward worse survival [70]. Functionally, SPHK1/S1P signaling exerts multiple pro-tumor effects: it promotes cell proliferation and survival by inhibiting apoptosis, induces EMT, and enhances treatment resistance [35, 59, 60, 71]. For example, high S1P levels can disrupt cell adhesion by downregulating E-cadherin, thereby facilitating tumor invasion [72]. This is consistent with our observation that SPHK1 inhibition led to a reversal of EMT markers [59, 60, 73]. In parallel, SPHK1 is also considered an important mediator of immune evasion. Previous studies have shown that SPHK1 can upregulate PD-L1 via MTA3, allowing tumor cells to escape T-cell attack, and that high SPHK1 expression in tumor-associated macrophages can induce CD8^+^ T-cell exhaustion [74, 75]. Similarly, our immune correlation analyses showed that gastric tumors with high SPHK1 expression tended to have increased infiltration of dendritic cells and neutrophils, but no corresponding increase in effector T cell infiltration, suggesting that SPHK1 may shape an immunosuppressive niche dominated by myeloid cells [76]. This observation is highly consistent with previous findings that SPHK1 fosters immunosuppression and CD8^+^ T-cell dysfunction in mouse models.

Given the central role of SPHK1 in metabolic reprogramming and immune escape, it represents a promising therapeutic target [30]. Several SPHK1 inhibitors have already been developed and have entered clinical evaluation. Selective SPHK1 inhibitors, such as PF-543, can reduce the expression of immunosuppressive factors in the tumor microenvironment, thereby reversing resistance to immune checkpoint blockade [77]. In animal models, combining SPHK1 inhibition with anti-PD-1 immunotherapy significantly suppressed tumor metastasis and prolonged survival [78, 79]. Similarly, experimental inhibition of SPHK1 has been shown to reverse tumor cell PD-L1 upregulation-mediated immune evasion, thereby improving the efficacy of anti-PD-1 therapy [79]. These studies suggest that targeting SPHK1 in gastric cancer could inhibit tumor progression and enhance the efficacy of immunotherapy. Recent reviews have emphasized sphingolipid metabolism as a emerging target for immunotherapy, potentially opening new therapeutic avenues. For example, recent evidence suggests that inhibition of sphingolipid metabolism can not only directly suppress tumor cells, but also improve the tumor microenvironment and enhance immune responses, providing a theoretical basis for future combination treatment strategies.

The central findings of this study are supported by multiple complementary lines of evidence, including bioinformatic analyses, SMG-based molecular subtyping, spatial transcriptomic characterization, and in vitro functional experiments. Collectively, these approaches provide a coherent framework supporting the role of sphingolipid metabolic remodeling, particularly aberrant SPHK1 activation, in gastric cancer progression and immune microenvironment regulation. Nevertheless, several limitations should be acknowledged. Although the current findings are supported by convergent evidence, experimental validation remains incomplete. In particular, the observed association between high SPHK1 expression and increased neutrophil/dendritic-cell infiltration remains primarily correlative, and additional functional studies, such as migration assays using conditioned media from SPHK1-modulated gastric cancer cells, will be necessary to determine whether SPHK1 directly contributes to myeloid-cell recruitment. Additional in vivo studies would further strengthen the mechanistic and translational significance of these findings. Future studies should therefore incorporate well-designed animal models and larger clinical cohorts to further validate the associations between SMG markers, prognosis, and immune response and to assess the therapeutic potential of SPHK1 inhibition in gastric cancer.

## Data Availability

All data needed to evaluate the conclusions of this study are present in the article. Additional data or materials related to this work are available from the corresponding author upon reasonable request.
